# Dihydrotestosterone is a predictor for mortality in males with community-acquired pneumonia: results of a 6-year follow-up study

**DOI:** 10.1186/s12931-018-0947-0

**Published:** 2018-12-04

**Authors:** Seline Zurfluh, Manuela Nickler, Manuel Ottiger, Christian Steuer, Alexander Kutz, Mirjam Christ-Crain, Werner Zimmerli, Robert Thomann, Claus Hoess, Christoph Henzen, Luca Bernasconi, Andreas Huber, Beat Mueller, Philipp Schuetz, for the proHOSP study group, Ursula Schild, Katharina Regez, Rita Bossart, Robert Thomann, Claudine Falconnier, Marcel Wolbers, Stefanie Neidert, Thomas Fricker, Claudine Blum, Thomas Bregenzer, Claus Hoess, Heiner C. Bucher, Fabian Mueller, Jeannine Haeuptle, Roya Zarbosky, Rico Fiumefreddo, Melanie Wieland, Charly Nusbaumer, Andres Christ, Roland Bingisser, Kristian Schneider, Brigitte Walz, Verena Briner, Dieter Conen, Andreas Huber, Jody Staehelin, Chantal Bruehlhardt, Ruth Luginbuehl, Agnes Muehlemann, Ineke Lambinon, Werner Zimmerli, Max Zueger

**Affiliations:** 1 Medical University Department, Division of General Internal and Emergency Medicine, Kantonsspital Aarau, Aarau, Switzerland; 2 ETH Zürich, Zürich, Switzerland; 3 Department of Internal Medicine, Division of Endocrinology, Diabetes and Clinical Nutrition, University Hospital Basel, Basel, Switzerland; 4 Basel University Medical Clinic Liestal, Liestal, Switzerland; 5 Department of Internal Medicine, Bürgerspital Solothurn, Solothurn, Switzerland; 6 Department of Internal Medicine, Kantonsspital Münsterlingen, Münsterlingen, Switzerland; 7 Department of Internal Medicine, Kantonsspital Lucerne, Lucerne, Switzerland; 8 Department of Laboratory Medicine, Kantonsspital Aarau, Aarau, Switzerland

**Keywords:** Community-acquired pneumonia, Adrenal hormones, Dihydrotestosterone, Mortality prediction

## Abstract

**Background:**

Adrenal hormone metabolite levels are altered in acute illnesses such as community-acquired pneumonia (CAP). Our aim was to investigate associations of sex and mineralocorticoid hormone metabolites with short- and long-term mortality and severity of CAP in male and female patients.

**Methods:**

We prospectively followed 285 patients (60.4% male, mean age 71 years) with CAP from a previous multicenter trial. At baseline, levels of different metabolites of sex hormones and mineralocorticoids were measured by liquid chromatography coupled to tandem mass spectrometry. We calculated Cox regression models adjusted for age and comorbidities.

**Results:**

All-cause mortality was 5.3% after 30 days and increased to 47.4% after 6 years. In males, high levels of dihydrotestosterone were associated with higher 6-year mortality (adjusted HR 2.84, 95%CI 1.15–6.99, *p = 0.023*), whereas high levels of 17-OH-progesterone were associated with lower 6-year mortality (adjusted HR 0.72, 95%CI 0.54–0.97, *p = 0.029*). Testosterone levels in males correlated inversely with inflammatory markers (CRP rho = − 0.39, *p < 0.001*; PCT rho = − 0.34, *p < 0.001*) and disease severity as assessed by the Pneumonia severity index (PSI) (rho = − 0.23, *p = 0.003*). No similar association was found for female patients.

**Conclusion:**

Whereas in males with CAP, sex and mineralocorticoid hormone metabolite levels correlated with inflammation, disease severity and long-term survival, no similar association was found for females. Further study of sex and mineralocorticoid hormones in acute illness could generate predictive signatures with implementation in clinical practice.

## Background

Community-acquired pneumonia (CAP) is the third leading cause of death worldwide [1]. Understanding factors that predict short-term mortality in CAP has been a research priority in recent years. As a result, clinical risk scores such as the pneumonia severity index (PSI) and prognostic blood markers such as pro-adrenomedullin (proADM) and procalcitonin (PCT) were found to correlate with short-term mortality and to be helpful for risk stratification of patients. Prognostic information about the expected short-term follow-up may help physician to make more rational decisions regarding inpatient or outpatient treatment. Less research, however, has focused on prognostic factors for prediction of long-term outcome in CAP.

Still, patients surviving an initial CAP episode are at increased risk for death and recurrent infections within the subsequent years. This may be explained by the fact that CAP is a surrogate for poor general condition and severe comorbidities. Interestingly, a more pronounced systemic inflammatory host response was shown to be associated with better long-term outcome [2]. Among other factors, an appropriate activation of the hypothalamic-pituitary-adrenal axis (HPA) is pivotal during acute illness such as CAP. Consecutively, the adrenal gland produces a variety of glucocorticoid, mineralocorticoid and sexual hormones. Different studies have shown a correlation of cortisol levels with severity, short- and long-term outcome in CAP and sepsis [3–8]. An independent association of aldosterone levels with survival-time in septic shock in canine bacterial sepsis has also been shown [6]. In addition, sex hormones are altered in infection and have modulatory function to the immune system [9]. Although the pathophysiology regarding the function of DHEA/-S during infection is still not conclusively understood, there seems to be an important link to survival [10]. Yet, results have been inconsistent with some studies reporting an association between low DHEA/-S levels and long-term mortality, especially in elderly males [10–16]. Although low testosterone levels are associated with higher long-term mortality in older males, it remains unclear if low testosterone levels are just surrogates of poor health status [17, 18] or if there is a causal link [17]. So far, most studies have focused on cortisol and DHEA/-S as marker of adrenal activation in sepsis and CAP. Data regarding other sex and mineralocorticoid hormone metabolites are missing. The aim of this study was to analyze different sex and mineralocorticoid hormone metabolites in CAP regarding their association with short- and long-term mortality, disease severity and inflammation markers.

## Methods

### Study design

We did a secondary analysis of data from a previous prospective, randomized, controlled multicenter trial conducted at six Swiss secondary or tertiary hospitals between October 2006 and March 2008 [19]. The aim of the initial trial was to evaluate efficacy and safety of PCT-guided antibiotic therapy in patients with lower respiratory tract infections (LRTI) [19]. The study protocol was approved by the ethics committees of the University of Basel as well as by all local ethics committees and has been published elsewhere [20]. All included patients provided informed consent for the initial trial as well as agreement to use their data anonymized for future secondary analysis.

### Study population

Inclusion criteria were age ≥ 18 years and a final diagnosis of CAP, defined as LRTI with an infiltrate on the chest x-ray [20]. Patients were excluded if they were incapable to give informed consent due to language restriction or severe dementia. Furthermore, exclusion criteria of the initial trial contained active intravenous drug use, severe immunosuppression, and life-threatening medical comorbidities with possibility of leading to imminent death, hospital-acquired pneumonia or long-term antibiotic treatment due to chronic infection.Clinical and biochemical data were assessed on admission and throughout time of hospitalization. Baseline characteristics included demographics, medical history, vital signs, comorbidities (identification through medical chart review or patient self-report), laboratory values, chest x-ray, and medication. Disease severity was assessed by commonly used risk score such as Pneumonia severity index (PSI), CURB-65 score and qSOFA at admission [21].From the initial study population with 1359 LRTI patients, 925 patients had CAP and 285 of these CAP patients had leftover serum samples for measurement of metabolomic markers.

### Steroid hormone analysis

Within the initial trial, blood serum samples were collected on admission and stored at − 80 °C for later measurements of different biomarkers. In our samples, we measured the following hormone metabolites; aldosterone, progesterone, OH-progesterone, DHEA, DHEA-S, testosterone, androstenedione, and dihydrotestosterone. After internal validation studies, concentration of these metabolites was determined using a commercially available kit (MassChrom Steroids; Chromsystems, Munich, Germany). The analysis was performed using the UltiMate 3000 ultra-high-performance liquid chromatography (UHPLC) system (Thermo Fisher Scientific, San Jose CA, USA) coupled to an AB Sciex 5500 quadrupole mass spectrometer (AB Sciex, Darmstadt, Germany). The Turbo V ion source (AB Sciex) was operated in positive electrospray ionization mode. The targeted screening method employed the multiple reaction monitoring mode of operation using two transitions for each analyte. Prior to injection into the UHPLC system, serum samples were subjected to a complex process of reversed phase 96-well solid-phase extraction, purification, and concentration steps as described in the MassChrom Steroids user’s manual. Quantification of selected metabolites was achieved by reference to appropriate internal standards. Concentrations of all analyzed metabolites were reported in nanomole per liter.

### Main outcome measurements

The primary outcome was defined as 6-year all-cause mortality. As secondary outcomes, we reported mortality at further time-points (at day 30, 60, 90, 180, 240 and 300, as well as after 1, 2 and 3 years).Outcome validation was performed by blinded, structured telephone interviews at day 30, 180 and 540 after admission, as well as after a median of 6.1 years (IQR 5.6–6.5). Initially, the patient or his household members were contacted and if they were not available the general practitioner was contacted to verify vital status.

### Statistical analysis

Statistical analyses were performed using STATA 12.1 Software (StataCorp, College Station, TX, USA). Testing was two-tailed, significance level was defined as *p*-value < 0.05. In descriptive statistics, continuous variables are expressed as median with IQR, categorical variables as counts and frequency. Two-group comparison was done by Wilcoxon rank-sum test, frequency comparison by Chi-square test. Association between hormone levels and all-cause mortality at different time-points was assessed by multivariate Cox regression analysis; results are reported as hazard ratios (HR) with 95% confidence interval (CI). Because of skewed distribution of hormone levels, we log-transformed levels with a base of 10 before entering the values in regression analysis. Therefore, HRs are equivalent to a tenfold increase in hormone levels. Multivariate models were adjusted for predefined factor expected to influence mortality or hormone levels, respectively, namely age and comorbidities (coronary heart disease, cerebrovascular insult, chronic renal failure, neoplastic disease). Analyses were further stratified by gender. Correlation analyses of hormone levels with inflammation markers was done by Spearman’s rank correlation. Multigroup comparisons were calculated by Kruskal-Wallis test.

## Results

### Characteristics of the study population

From a total of 285 patients included, 15 (5.3%) died within 30 days and a total of 135 (47.4%) died during the 6-year follow up. In the male cohort, 9 (5.2%) out of 172 died within 30 days, 92 (53.5%) within 6 years. The median age of the entire cohort was 71 years, 60.4% of the patients were male. Most frequent PSI class was IV (36.5%), and CURB-65 score II (28.8%). Patients had an important burden of comorbidities with 23.5% (*n* = 28) of patients having chronic renal failure, 20.7% (*n* = 20) having coronary artery disease, 19.3% (*n* = 55) having diabetes mellitus, 15.4% (*n* = 44) having congestive heart failure, and 13.3% (*n* = 38) having neoplastic disease. Finally, we had available hormone levels for aldosterone in 214 patients (male *n* = 124, female *n* = 90), for progesterone in 232 patients (male *n* = 143, female *n* = 89), for 17-OH-progesterone in 255 patients (male *n* = 160, female *n* = 95), for DHEA in 76 patients (male *n* = 42, female *n* = 34), for DHEA-S in 67 patients (male n = 42, female *n* = 25), for testosterone in 280 patients (male *n* = 169, female *n* = 111), for androstenedione in 281 patients (male n = 169, female *n* = 112), and for dihydrotestosterone in 79 patients (male *n* = 55, female *n* = 24). Table 1 shows additional Baseline characteristics for the entire cohort, as well as stratified by gender and by the primary endpoint.

**Table 1:** Baseline characteristics overall and stratified by gender and 6-year vital status in CAP

Characteristics	Entire cohort (*N* = 285)	MALE (*N* = 172)			FEMALE (*N* = 113)		
		6-year vital status					
		Survivors (*n* = 80)	Non-survivors (*n* = 92)	*P value*	Survivors (*n* = 70)	Non-survivors (*n* = 43)	*p* value
*Demographics*							
Age	71 [57, 81]	65 [46.5, 74.5]	78 [70, 84]	**< 0.001**	62 [43, 75]	79 [72, 85]	**< 0.001**
Male	172 (60.4%)						
*CAP characteristics*							
PSI class							
I	32 (11.2%)	12 (15%)	0 (0%)	**< 0.001**	18 (26%)	2 (5%)	**0.004**
II	55 (19.3%)	22 (28%)	5 (5%)	**< 0.001**	22 (31%)	6 (14%)	**0.037**
III	52 (18.2%)	16 (20%)	16 (17%)	0.66	13 (19%)	7 (16%)	0.76
IV	104 (36.5%)	23 (29%)	45 (49%)	**0.007**	15 (21%)	21 (49%)	**0.002**
V	42 (14.7%)	7 (9%)	26 (28%)	**0.001**	2 (3%)	7 (16%)	**0.011**
CURB-65 score							
0	63 (22.1%)	24 (30%)	6 (7%)	**< 0.001**	27 (39%)	6 (14%)	**0.005**
I	67 (23.5%)	20 (25%)	21 (23%)	0.74	21 (30%)	5 (12%)	**0.024**
II	82 (28.8%)	22 (28%)	29 (32%)	0.56	11 (16%)	20 (47%)	**< 0.001**
III	57 (20.0%)	11 (14%)	27 (29%)	**0.014**	10 (14%)	9 (21%)	0.36
I*V*/V	16 (5.6%)	3 (4%)	9 (10%)	0.12	1 (1%)	3 (7%)	0.12
*Comorbidities* ^a^							
Coronary heart disease	59 (20.7%)	11 (14%)	34 (37%)	**< 0.001**	5 (7%)	9 (21%)	**0.031**
Congestive heart failure	44 (15.4%)	4 (5%)	25 (27%)	**< 0.001**	3 (4%)	12 (28%)	**< 0.001**
Cerebrovascular insult	28 (9.8%)	4 (5%)	15 (16%)	**0.018**	5 (7%)	4 (9%)	0.68
PAOD	17 (6.0%)	5 (6%)	8 (9%)	0.55	2 (3%)	2 (5%)	0.62
Chronic renal failure	67 (23.5%)	12 (15%)	30 (33%)	**0.007**	7 (10%)	18 (42%)	**< 0.001**
Diabetes mellitus	55 (19.3%)	14 (18%)	23 (25%)	0.23	8 (11%)	10 (23%)	0.095
Neoplastic disease	38 (13.3%)	9 (11%)	23 (25%)	**0.021**	3 (4%)	3 (7%)	0.54
*Clinical history*							
Fever	185 (65.1%)	68 (85%)	48 (53%)	**< 0.001**	45 (64%)	24 (56%)	0.37
Chills	87 (34.0%)	31 (42%)	23 (29%)	0.086	27 (42%)	6 (15%)	**0.006**
Glucocorticoid pretreatment	22 (7.9%)	2 (3%)	13 (14%)	**0.008**	3 (4%)	4 (10%)	0.27
*Clinical findings*							
Confusion	20 (7.9%)	4 (5%)	13 (16%)	**0.034**	0 (0%)	3 (8%)	**0.022**
Body temperature, °C	38 [37.2, 38.8]	38.4 [37.5, 39]	38 [37, 38.8]	0.090	37.9 [37.2, 38.6]	37.6 [36.9, 38.6]	0.21
Breath rate, beaths/min.	20 [16, 25]	20 [16, 24]	24 [18, 28]	**0.009**	20 [17.00, 24.00]	22 [17, 28]	0.14
Heart rate, beats/min.	94 [82, 105]	92 [86, 105.5]	95.5 [80, 105]	0.86	95 [82, 109]	93 [82, 100]	0.28
SBP, mmHg	130 [117, 148]	130.5 [121, 150]	128.5 [104, 150]	0.077	131 [117, 145]	133 [118, 145]	0.96
Arterial pH	7.46 [7.42, 7.49]	7.46 [7.44, 7.49]	7.44 [7.41, 7.48]	0.095	7.46 (7.43, 7.50)	7.45 (7.40, 7.50)	0.11
qSOFA (≥ 2)	25 (10.6%)	2 (3%)	18 (25%)	**< 0.001**	0 (0%)	5 (14%)	**0.003**
*Admission laboratory findings*							
CRP, mg/l	132 [65, 252]	145 [78, 240]	110 [55, 252]	0.17	148[105, 284]	121 [62, 234]	0.13
PCT, mcg/l	0.48 [0.16, 3.20]	0.42 [0.19, 3.56]	0.51 [0.16, 2.60]	0.48	0.72 [0.12, 5.58]	0.32 [0.16, 1.50]	0.52
Progesterone, nmol/l	0.69 [0.44, 1.43]	0.69 [0.46, 1.57]	0.66 [0.37, 1.06]	0.26	0.88 [0.56, 2.62]	0.73 [0.52, 1.09]	0.13
17-OH-Progesterone, nmol/l	1.74 [0.87, 3.35]	1.88 [1.08, 3.32]	1.67 [0.96, 3.36]	0.51	1.67 [0.54, 3.50]	1.55 [0.63, 3.18]	0.94
Aldosterone, nmol/l	0.06 [0.03, 0.20]	0.07 [0.04, 0.22]	0.05 [0.03, 0.18]	0.18	0.05 [0.03, 0.17]	0.08 [0.04, 0.19]	0.43
DHEA, nmol/l	13.43 [7.73, 209.55]	39.74 [8.8, 409.4]	7.67 [6.27, 142.2]	0.071	21.84 [9.90, 330.01]	11.47 [6.78, 26.52]	0.18
DHEA-S, nmol/l	2280.09 [836.72, 3660.99]	3975.42 [2837.7, 7960.95]	1193.58 [647.62, 2559.29]	**< 0.001**	2317.52 [1537.38, 3660.99]	911.44 [379.14, 2506.82]	0.081
Androstenedione, nmol/l	2.78 [1.36, 4.58]	2.86 [1.29, 4.33]	2.33 [1.24, 4.35]	0.44	3.18 [1.75, 6.24]	2.80 [1.18, 4.78]	0.11
Testosterone, nmol/l	1.63 [0.49, 4.68]	4.36 [1.98, 6.22]	3.29 [2.04, 6.39]	0.62	0.50 [0.29, 0.70]	0.34 [0.24, 0.56]	0.13
Dihydrotestosterone, nmol/l	1.31 [0.26, 2.83]	0.91 [0.22, 2.16]	1.59 [1.07, 3.42]	0.11	0.84 [0.42, 2.83]	1.69 [0.18, 3.09]	0.93

Data are presented as median [IQR] or number (percentage); *p* values are considered statistically significant at *p* < 0.05. Bold values indicate statistical significance. *CAP* community-acquired pneumonia, *CRP* C-reactive protein, *CURB-65* ‘confusion’, ‘urea > 7 mmol/L’, ‘respiratory rate > 30/min’, ‘blood pressure systolic < 90 mmHg or diastolic < 60 mmHg’, ‘age ≥ 65 years’; *DHEA* dihydroepiandrosterone, *DHEA-S* dihydroepiandrosterone sulfate, *IQR* interquartile range, *PAOD* peripheral artery occlusive disease, *PCT* procalcitonin, *PSI* pneumonia severity index, *SBP* systolic blood pressure, *qSOFA* quick sequential organ failure assessment

^a^Comorbidities were identified based on medical records or patient report

### Time-dependent association between admission hormone metabolite levels and mortality

Associations between admission sex and mineralocorticoid hormone levels and all-cause mortality at the different time points are shown separated for males (Table 2*)* and females (Table 3*)*. Regarding sex hormone metabolites, our results showed that in males high initial levels of dihydrotestosterone were associated with increased 6-year mortality (adj. HR 2.84, 95%CI 1.15–6.99, *p = 0.023*), we also found this association in the entire cohort adjusted for gender (adj. HR 1.78, 95%CI 1.03–3.09, *p = 0.040;* Table 4 *in* *Appendix*). For females, this association was not significant. High initial levels of progesterone and 17-OH-progesterone were significantly associated with improved survival at 3 years and 6 years (adj. HR 0.61, 95%CI 0.39–0.97, *p = 0.037 and* adj. HR 0.75, 95%CI 0.57–0.98, *p = 0.034*). This association of 17-OH-progesterone could also be found in the male cohort, but not in females. The other sex hormone metabolites and aldosterone showed no significant association with short- or long-term mortality.

**Table 2:** Association of admission sex and mineralocorticoid hormone metabolite levels with short- and long-term all-cause mortality in **males** with CAP

Men (N = 172)	All-cause mortality timepoint					
	30 days		3 years		6 years	
	*HR (95%CI)*	*p value*	*HR (95%CI)*	*p value*	*HR (95%CI)*	*p value*
*Progesterone*						
Cox regression analyses	0.66 (95%CI 0.11–3.83)	*p* = 0.643	0.66 (95%CI 0.34–1.31)	*p* = 0.239	0.69 (95%CI 0.40–1.18)	*p* = 0.178
*17-OH-Progesterone*						
Cox regression analyses	0.72 (95%CI 0.35–1.48)	*p* = 0.369	0.66 (95%CI 0.47–0.92)	***p*** **= 0.015**	0.72 (95%CI 0.54–0.97)	***p*** **= 0.029**
*Aldosterone*						
Cox regression analyses	1.91 (95%CI 0.55–6.63)	*p* = 0.310	1.25 (95%CI 0.74–2.12)	*p* = 0.410	0.89 (95%CI 0.59–1.33)	*p* = 0.565
*DHEA*						
Cox regression analyses			0.16 (95%CI 0.01–3.79)	*p* = 0.255	0.67 (95%CI 0.35–1.27)	*p* = 0.221
*DHEA-S*						
Cox regression analyses	0.57 (95%CI 0.01–25.17)	*p* = 0.768	0.69 (95%CI 0.20–2.42)	*p* = 0.562	0.55 (95%CI 0.18–1.67)	*p* = 0.293
*Androstenedione*						
Cox regression analyses	5.46 (95%CI 0.85–35.12)	*p* = 0.074	0.59 (95%CI 0.34–0.99)	***p*** **= 0.049**	0.65 (95%CI 0.40–1.05)	*p* = 0.081
*Testosterone*						
Cox regression analyses	0.69 (95%CI 0.16–2.94)	*p* = 0.613	1.36 (95%CI 0.73–2.52)	*p* = 0.328	1.04 (95%CI 0.64–1.72)	*p* = 0.865
*Dihydrotestosterone*						
Cox regression analyses	6.08 (95%CI 0.15–254.59)	*p* = 0.344	2.0 (95%CI 0.64–6.27)	*p* = 0.236	2.84 (95%CI 1.15–6.99)	***p*** **= 0.023**

Data for multivariate Cox regression models are presented as HR (95% CI), p value; *p* values are considered statistically significant at *p* < 0.05. Bold values indicate statistical significance. All hormone metabolite levels were log-transformed and thus the HR corresponds to a 10-fold increase in these levels. *CI* confidence interval, *DHEA* dehydroepiandrosterone, *DHEA-S* dehydroepiandrosterone sulfate, *HR* hazard ratio

The multivariate model is adjusted for age and comorbidities (coronary artery disease, cerebrovascular disease, chronic kidney disease, neoplastic disease)

**Table 3:** Association of admission sex and mineralocorticoid hormone metabolite levels with short- and long-term all-cause mortality in **females** with CAP

Females (N = 113)	All-cause mortality timepoint					
	30 days		3 years		6 years	
	*HR (95%CI)*	*p value*	*HR (95%CI)*	*p value*	*HR (95%CI)*	*p value*
*Progesterone*						
Cox regression analyses	0.34 (95%CI 0.004–25.46)	*p* = 0.621	0.24 (95%CI 0.08–0.71)	***p*** **= 0.010**	0.44 (95%CI 0.17–1.14)	*p* = 0.093
*17-OH-Progesterone*						
Cox regression analyses	0.55 (95%CI 0.09–3.46)	*p* = 0.528	0.99 (95%CI 0.45–2.19)	*p* = 0.979	0.98 (95%CI 0.50–1.93)	*p* = 0.963
*Aldosterone*						
Cox regression analyses	0.29 (95%CI 0.04–2.14)	*p* = 0.226	0.64 (95%CI 0.30–1.39)	*p* = 0.263	0.81 (95%CI 0.43–1.53)	*p* = 0.516
*DHEA*						
Cox regression analyses			1.98 (95%CI 0.33–11.97)	*p* = 0.456	1.44 (95%CI 0.30–6.91)	*p* = 0.645
*DHEA-S*						
Cox regression analyses			0.69 (95%CI 0.22–2.19)	*p* = 0.529	0.55 (95%CI 0.23–1.31)	p = 0.178
*Androstenedione*						
Cox regression analyses	1.41 (95%CI 0.31–6.38)	*p* = 0.652	1.03 (95%CI 0.58–1.83)	*p* = 0.925	1.10 (95%CI 0.68–1.79)	*p* = 0.695
*Testosterone*						
Cox regression analyses	0.92 (95%CI 0.29–2.92)	*p* = 0.887	0.81 (95%CI 0.40–1.67)	*p* = 0.574	0.91 (95%CI 0.48–1.70)	*p* = 0.758
*Dihydrotestosterone*						
Cox regression analyses			1.78 (95%CI 0.60–5.25)	*p* = 0.296	1.20 (95%CI 0.56–2.59)	*p* = 0.638

Data for multivariate Cox regression models are presented as HR (95% CI), *p* value; *p* values are considered statistically significant at *p* < 0.05. Bold values indicate statistical significance. All hormone metabolite levels were log-transformed and thus the HR corresponds to a 10-fold increase in these levels. *CI* confidence interval, *DHEA* dehydroepiandrosterone, *DHEA-S* dehydroepiandrosterone sulfate, *HR* hazard ratio

The multivariate model is adjusted for age and comorbidities (coronary artery disease, cerebrovascular disease, chronic kidney disease, neoplastic disease)

### Association between admission hormone metabolite levels and severity of CAP

**Fig. 1:**
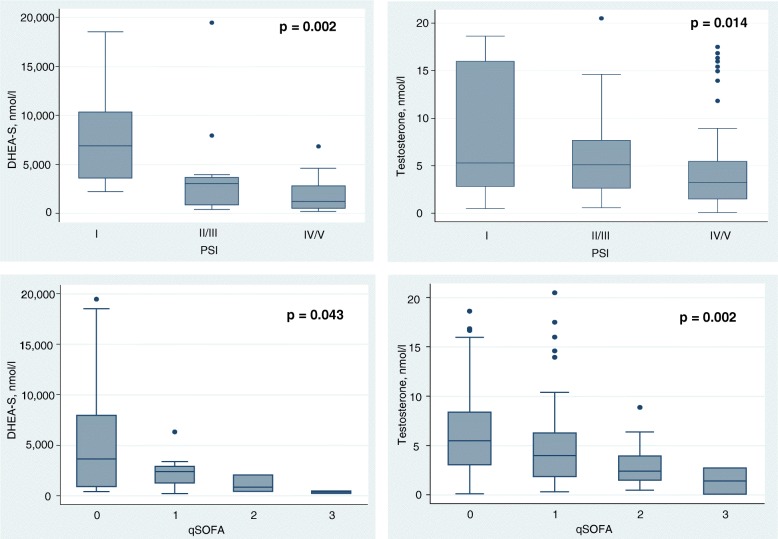
Admission DHEA-S and testosterone levels with according disease severity (PSI and qSOFA) in males with CAP. Data are represented as median and IQR, with scatter plots representing each value. *P* values are determined by Kruskal-Wallis test and considered statistically significant at *p* < 0.05. Bold values indicate statistical significane. CAP, community-aquired pneumonia; DHEA-S, dihydroepiandrosterone-sulfate; PSI, pneumonia severity index; qSOFA, quick sequential organ failure assessment

We also analyzed the association of hormone levels and initial severity of CAP as assessed by inflammatory biomarkers and clinical risk scores. In males, DHEA-S and testosterone levels correlated inversely with disease severity; DHEA-S (PSI: rho = − 0.55, *p < 0.001*, qSOFA: *p = 0.043*), testosterone (PSI: rho = − 0.23, *p = 0.003*, qSOFA: *p = 0.002*). Box plots showing DHEA-S and testosterone levels according to PSI class and qSOFA are presented in Fig. 1.

### Correlation of admission hormone levels and inflammatory markers

**Fig. 2:**
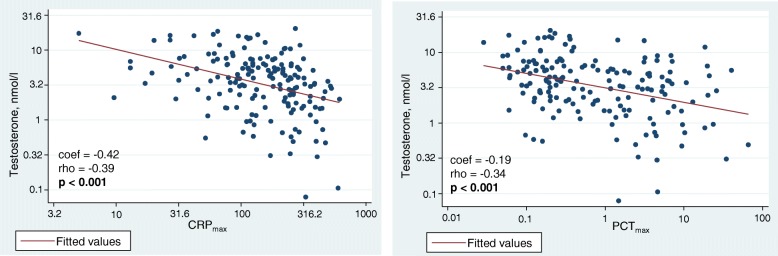
Correlation of admission testosterone levels with acute inflammatory markers (CRP and PCT) in **males** with CAP. Data are presented with scatterplots showing all values (blue), overlaid by linear fit lines (red). We used admission hormone metabolite levels and peak values of CRP and PCT. Correlation analyses were performed by Spearman’s rank correlation (rho; *p*-value). *p* < 0.05 is considered statistically significant; bold values indicate statistical significance. We used multivariate linear regression models to calculate regression coefficients (coef). CAP, community-acquired pneumonia; CRP, c-reactive protein; PCT, procalcitonin. *Multivariate model is adjusted for age and comorbidities (coronary artery disease, cerebrovascular disease, chronic kidney disease, neoplastic disease)

In males, there was an inverse correlation of testosterone levels with acute inflammatory markers, namely CRP (rho = − 0.39, *p* < 0.001) and PCT (rho = − 0.34, *p* < 0.001). These correlations are shown as scatterplots in Fig. 2. The other hormone metabolites did not correlate with inflammatory markers in males or females.

## Discussion

The main findings of our analyses of sex and mineralocorticoid hormone metabolites regarding their prognostic value in CAP patients over a follow-up period of 6 years are threefold. First, in males higher serum levels of dihydrotestosterone on admission were associated with higher long-term mortality. Secondly, in males serum testosterone levels correlated inversely with disease severity and inflammation markers. Third, high initial serum levels of 17-OH-progesterone were associated with better long-term outcome in male patients. Regarding sex hormones, our results show a significant association of higher initial dihydrotestosterone levels with increased long-term mortality in males. A modulatory function of sex hormones to the immune system has been described, although exact mechanisms are not completely understood [9, 22, 23]. Testosterone levels decline with age, chronic illness and obesity. In community-based studies of elderly males associations of low (dihydro-)testosterone and increased all-cause and cardiovascular mortality have been described [17, 24–26]. Causality of this association is still unclear, since low levels of testosterone could either be a marker of poor health or testosterone deficiency itself could increase the cardiovascular risk [17]. However, there is also literature reporting no association between testosterone levels and mortality or cardiovascular disease, respectively [27]. Other studies indicate a nonlinear association and postulate an optimal range of testosterone levels [28, 29]. Considering this, our results, showing an association of high dihydrotestosterone levels and increased long-term mortality in CAP, seem counterintuitive at first. But importantly, several studies have shown temporary hypogonadism being an appropriate and pivotal reaction in acute inflammation because highest priority is survival, not reproduction [18, 30, 31]. Therefore, it is likely that low dihydrotestosterone levels in this cohort of CAP patients are not solely a sign of poor general health, but more a sign of an adequate suppression of the gonadal activity in acute illness. Furthermore, our results showed that in males testosterone and DHEA-S levels inversely correlate with inflammatory markers (CRP and PCT), which supports the hypothesis that with increased disease severity and a more pronounced inflammatory response sex hormones are suppressed [18]. Suppression of dihydrotestosterone indicates therefore a more pronounced inflammatory response, which has been shown to be beneficial regarding long-term survival for patients after surviving CAP [2]. In addition, initial high serum levels of 17-OH-progesterone were associated with significant better long-term survival in males and in the entire cohort. 17-OH-progesterone is a precursor of the stress hormone cortisol; thus, it is likely that higher serum levels of 17-OH-progesterone reflect a more pronounced stress response with cortisol production, which has already been shown to be beneficial regarding long-term survival in CAP patients [8].We did not find these associations of dihydrotestosterone or 17-OH-progesterone with long-term mortality in females. The female population was significantly younger, had a longer life expectancy and 6-year mortality was significantly lower (38%) than in the male population (53%). This may have affected power in the female population and led to type II error. Furthermore, our results showed no association of the mineralocorticoid aldosterone with neither short-, long-term mortality nor disease severity. Strengths of this study are the well-defined cohort of CAP patients, the long median follow up-time of 6.1 years and the exact measurement of the hormone metabolites by liquid chromatography coupled to tandem mass spectrometry. In addition, the high event number of the primary endpoint (47.4%) leads to high statistical power.

As limitations of the study, the following points should be considered. First, this is a secondary analysis and therefore the initial trial was not designed with the intention to perform biomarker outcome studies. Numbers of some hormone metabolites, especially when stratified by gender, were small and therefore power to detect significant associations was limited. Time-point of blood sampling was not controlled - in fact blood samples were taken at the time of first contact when patients presented at emergency department. Thus, not considered circadian patterns are potential confounders. In addition, long storage of blood samples may have affected hormone metabolites, although in previous studies steroid hormone metabolites were found to be relatively stable when storage at ≤ − 80 °C [32–34]. Secondly, the study was performed at multiple hospitals in Switzerland with mainly Caucasian patients with CAP, therefore the results cannot unconditionally be applied to other geographical settings or other patient cohorts. Third, most included patients were elderly and results may not be generalizable to younger patients. Finally, this study is an observational study and therefore we cannot conclude any causal relationships.

## Conclusion

In males with CAP, lower initial serum levels of dihydrotestosterone were associated with favorable long-term survival. Furthermore, in males testosterone and DHEA-S levels inversely correlated with disease severity and inflammatory markers. In females, no association between sex hormone metabolites and outcome in CAP was found. Further research is needed to investigate causality of the found associations. Better understanding of sex hormone metabolites in acute illness could generate predictive signatures with implementation in clinical practice.

## Appendix

**Table 4:** Association of admission sex and mineralocorticoid hormone metabolite levels with short- and long-term all-cause mortality in CAP, entire cohort

Entire cohort (N = 285)	All-cause mortality timepoint					
	30 days		3 years		6 years	
	*HR (95%CI)*	*p value*	*HR (95%CI)*	*p value*	*HR (95%CI)*	*p value*
*Progesterone*						
Cox regression analyses	0.72 (95%CI 0.15–3.34)	*p* = 0.666	0.49 (95%CI 0.28–0.88)	***p*** **= 0.016**	0.61 (95%CI 0.39–0.97)	***p*** **= 0.037**
*17-OH-Progesterone*						
Cox regression analyses	0.73 (95%CI 0.39–1.36)	*p* = 0.324	0.70 (95%CI 0.52–0.96)	***p*** **= 0.027**	0.75 (95%CI 0.57–0.98)	***p*** **= 0.034**
*Aldosterone*						
Cox regression analyses	1.41 (95%CI 0.56–3.56)	*p* = 0.461	0.93 (95%CI 0.62–1.37)	*p* = 0.700	0.81 (95%CI 0.60–1.11)	*p* = 0.200
*DHEA*						
Cox regression analyses	45.73 (95%CI 0.01–295,289.2)	*p* = 0.393	0.46 (95%CI 0.17–1.25)	*p* = 0.126	0.64 (95%CI 0.36–1.21)	*p* = 0.119
*DHEA-S*						
Cox regression analyses	0.57 (95%CI 0.01–25.17)	*p* = 0.768	0.75 (95%CI 0.50–1.12)	*p* = 0.155	0.72 (95%CI 0.51–1.01)	*p* = 0.060
*Androstenedione*						
Cox regression analyses	2.93 (95%CI 0.74–11.62)	*p* = 0.126	0.79 (95%CI 0.56–1.12)	*p* = 0.180	0.90 (95%CI 0.67–1.21)	*p* = 0.478
*Testosterone*						
Cox regression analyses	0.80 (95%CI 0.30–2.15)	*p* = 0.658	1.10 (95%CI 0.71–1.71)	*p* = 0.656	1.03 (95%CI 0.71–1.51)	*p* = 0.867
*Dihydrotestosterone*						
Cox regression analyses	2.45 (95%CI 0.39–15.42)	*p* = 0.339	1.85 (95%CI 0.93–3.67)	*p* = 0.078	1.78 (95%CI 1.03–3.09)	***p*** **= 0.040**

Data for multivariate Cox regression models are presented as HR (95% CI), *p* value; *p* values are considered statistically significant at *p* < 0.05. Bold values indicate statistical significance. All hormone levels were log-transformed and thus the HR corresponds to a 10-fold increase in these levels. *CI* confidence interval, *DHEA* dehydroepiandrosterone, *DHEA-S* dehydroepiandrosterone sulfate, *HR* hazard ratio

The multivariate model is adjusted for age and comorbidities (coronary artery disease, cerebrovascular disease, chronic kidney disease, neoplastic disease)

## Abbreviations

adj: adjusted
CAP: community-acquired pneumonia
CI: confidence interval
CRP: C-reactive protein
CURB-65: ‘confusion’, ‘urea > 7mmol/L’, ‘respiratory rate > 30/min’, ‘blood pressure systolic < 90mmHg or diastolic < 60mmHg’, ‘age ≥ 65 years’
DHEA: dehydroepiandrosterone
DHEA-S: dehydroepiandrosterone sulfate
HPA: hypothalamic-pituitary-adrenal
HR: hazard ratio
IQR: interquartile range
LRTI: lower respiratory tract infection
PAOD: peripheral artery occlusive disease
PCT: procalcitonin
proADM: pro-adrenomedullin
PSI: pneumonia severity index
qSOFA: quick sequential organ failure assessment
SBP: systolic blood pressure
UHPLC: ultra-high-performance liquid chromatography
WBC: white blood cell
